# Large Bowel Obstruction Subsequent to Resected Lobular Breast Carcinoma: An Unconventional Etiology of Malignant Obstruction

**DOI:** 10.1155/2018/6085730

**Published:** 2018-06-13

**Authors:** Melissa Amberger, Nancy Presnick, Gerard Baltazar

**Affiliations:** ^1^Department of Surgery, SBH Health System, Bronx, NY, USA; ^2^New York Institute of Technology College of Osteopathic Medicine, New York Institute of Technology, Old Westbury, NY, USA; ^3^School of Medicine, City University of New York, New York, NY, USA

## Abstract

**Introduction:**

Breast cancer metastasis to the gastrointestinal tract is rare and mostly limited to case reports which recommend consideration of metastasis when breast cancer patients particularly those with invasive lobular carcinoma present with new gastrointestinal complaints.

**Presentation of case:**

We report a 50-year-old female who presented with gastrointestinal symptoms of nausea and vomiting determined to be the result of large bowel obstruction secondary to rectosigmoid metastasis and carcinomatosis of breast invasive lobular carcinoma. She was treated with diverting loop sigmoid colostomy for her large bowel obstruction.

**Discussion:**

Our case reflects the importance of gastrointestinal surveillance of patients with a history of breast cancer. Current National Comprehensive Cancer Network (NCCN) guidelines for stage I-II breast cancer suggest posttreatment lab and imaging evaluation for metastasis only if new symptoms present.

**Conclusion:**

We observed an unusually rapid disease progression, requiring evaluation of new gastrointestinal symptoms. Assessment for GI tract metastatic involvement should be done as early as progression to symptomatic disease can result in need for further invasive surgery in advanced stages of cancer.

## 1. Introduction

Gastrointestinal (GI) metastasis of breast carcinoma is rare [1–4]. A recent large study found only 1.55% of patients with metastatic breast cancer had involvement of the GI tract [3]. Autopsy findings, however, have shown a higher percentage of GI metastasis than clinical studies [2, 4]. When GI involvement does occur, the most common sites are the stomach and colon [5–8].

Information regarding breast cancer metastases to the GI tract is mostly limited to case reports, with the exception of a couple of large studies [3, 5]. These metastases often present as nonspecifically, as abdominal pain, nausea, vomiting, diarrhea, and changes in bowel habits [5, 6].

Interval between primary diagnosis and GI metastasis varies widely with an average of seven years but range of 0–30 years [5, 8, 9]. Metastasis can also occur synchronously with primary diagnosis [9–11]. Such variance presents a diagnostic challenge, particularly regarding timing and extent of metastatic workup in setting of nonspecific GI complaints.

Multiple studies have shown infiltrating lobular carcinoma more frequently metastasizes to the GI tract compared to infiltrating ductal carcinoma (IDC) [2, 8, 12–14]. Though IDC represents a majority of breast carcinoma cases with an incidence of 72.8%, ILC is the second most common ranging from 6 to 14% of breast cancer cases [5, 12, 13, 15–17].

Herein, we present a patient who developed colonic metastases within one year of ILC diagnosis and surgical treatment.

## 2. Presentation of Case

A 50-year-old female presented for second opinion of a small, slow-growing tumor under her right areola. In addition to this, she stated she had recently had a positive cervical Papanicolaou smear and was told she might have cervical cancer. She subsequently underwent bilateral diagnostic mammography revealing bilateral masses. She underwent core biopsy, which confirmed both masses to be infiltrating lobular carcinoma. Her cancer was noted to be estrogen receptor (ER) and progesterone receptor (PR) positive and human epidermal growth factor 2 (HER2) negative. Her past medical history was significant for active cocaine drug abuse. Due to her dependence on cocaine, her treatment course was complicated by recidivism of drug use and poor compliance with scheduled follow-up care.

Positron emission tomography (PET) and computerized tomography (CT) scans were obtained to evaluate for extent of disease given possible history of uterine cancer. Both were negative for distant metastasis. Consequently, she was diagnosed with left stage I ILC and right stage IIIA ILC. Two months later, after multiple missed follow-up appointments as well as missed scheduled surgery, the patient was admitted for a nonrelated medical complaint. She then underwent a right modified radical mastectomy and left total mastectomy with sentinel lymph node biopsy with tissue expander placement for breast reconstruction. Final pathology showed the left breast with a one-centimeter (cm) tumor, all margins and four nodes negative, and the right breast with a three cm tumor with focal small lymphovascular and perineural invasion, all margins and 17 nodes negative. The patient had an uncomplicated postoperative course; however, she did not complete recommended postoperative care including adjuvant chemotherapy treatment.

Eight months after initial presentation, outpatient colonoscopy was performed for abdominal pain. During the procedure, the colonoscope could not be passed beyond 15 cm due to stricture. Biopsy of the stricture was taken which proved pathologically to be a benign hyperplastic polyp.

One month after colonoscopy, she presented to the emergency department (ED) for abdominal pain. Abdominopelvic CT with oral contrast demonstrated small retroperitoneal lymph nodes determined to be potentially reactive or due to lymphoproliferative disorder. No evidence of acute small or large bowel disease was found.

**Figure 1:**
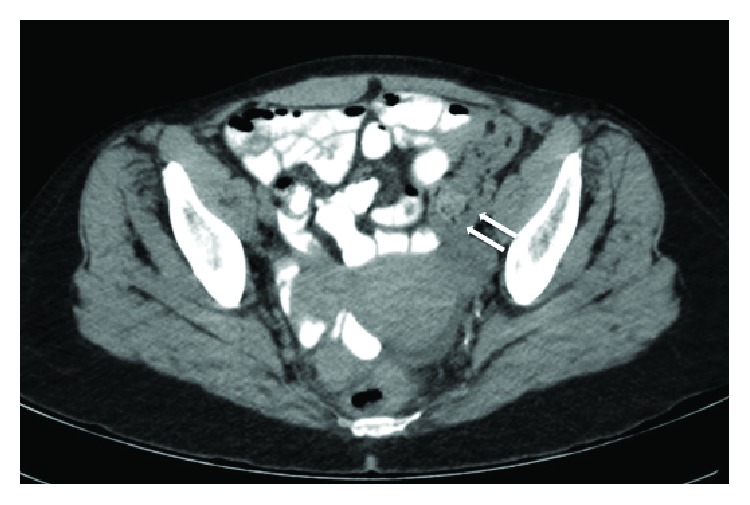
Sigmoid colonic wall thickening represented by white arrows near the area of stricture.

**Figure 2:**
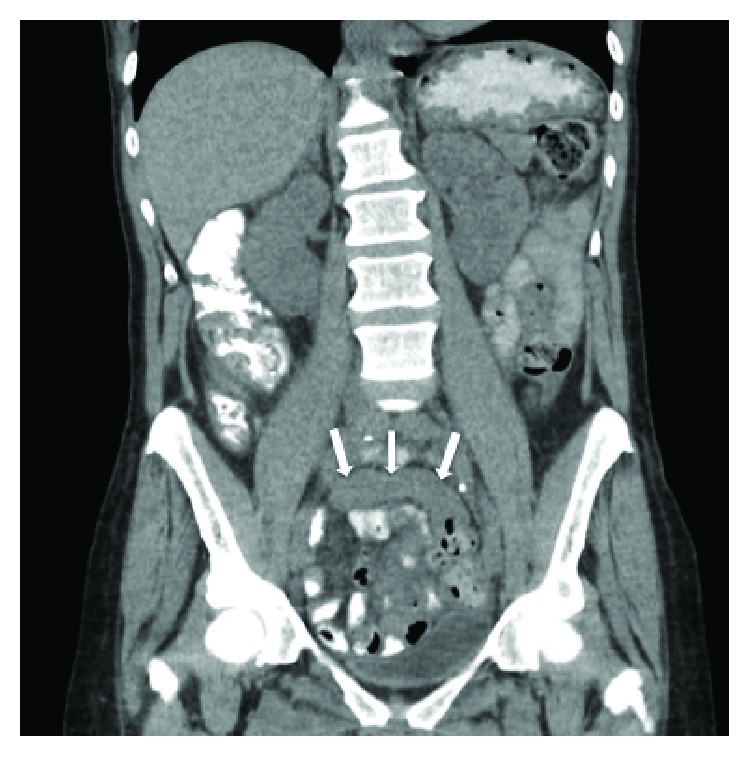
Rectosigmoid colonic wall thickening again demonstrated with white arrows at the site of stricture.

Two months after ED presentation, she returned to the ED complaining of nausea, vomiting, and abdominal pain. She had diffuse tenderness and voluntary guarding, but no rebound tenderness. No palpable masses were appreciated. Abdominopelvic CT showed diffuse colonic wall thickening and lymphadenopathy (Figures 1 and 2).

Due to suspicions of synchronous colonic malignancy, she was admitted for further investigation. On colonoscopy, a left-sided mass that was near completely obstructing prevented advancement of the colonoscopy beyond 20 cm. The colonic lumen was not easily identified, and extrinsic compression was severe. Biopsy again revealed a benign, hyperplastic polyp.

The following month, she agreed to exploratory surgery and was readmitted. She underwent rigid sigmoidoscopy followed by diagnostic laparoscopy. Intraoperative findings included a rectosigmoid stricturing likely representing metastasis and diffuse intra-abdominal carcinomatosis. Due to the inability to pass the rigid sigmoidoscope past the stricture and concern for metastatic involvement of the colon causing obstruction, a sigmoid loop colostomy was created. Peritoneal and omental biopsies were taken. Final postsurgical pathology confirmed metastatic invasive lobular breast carcinoma positive for ER, PR, cytokeratin-7 (CK7), and gross cystic disease fluid protein 15 (GCDFP15) and negative for cytokeratin-20 (CK20) and caudal type homeobox-2 (CDX-2). She refused further treatment for her cancer and was subsequently lost to follow-up.

## 3. Discussion

Colonic metastases from primary breast carcinoma are a rare entity without typical presentation and progression. Case reports suggest the importance of considering metastasis in patients with new GI symptoms and a history of breast cancer, especially ILC given a higher incidence of GI metastasis with ILC than IDC [2, 13, 14].

Colonic breast metastases may mimic a primary GI malignancy, complicating diagnosis and treatment [18, 19]. Consequently, in some cases, patients were initially misdiagnosed [5, 10].

Lau et al. describe a rectal lesion initially thought to be a primary rectal adenocarcinoma after colonoscopy. A magnetic resonance image (MRI) to stage the rectal mass was nondiagnostic, and biopsy suggested metastatic breast cancer. Hormone receptor status subsequently confirmed lobular breast carcinoma.

Matsuda et al. detail a diagnosis of primary, poorly differentiated rectal adenocarcinoma after endoscopic biopsy. After a poor response to therapy, further evaluation including immunostaining for CK7, estrogen receptor alpha, CK20, and E-cadherin changed the diagnosis to metastatic breast carcinoma.

Finally, McLemore et al. describe eight cases where diagnosis of metastatic breast cancer could not be made until exploratory laparotomy. Two of these patients had originally been diagnosed as having a primary GI adenocarcinoma.

Our report concurs with others who have suggested the importance of immunohistochemistry in making definitive diagnosis [4, 10, 11, 18, 20]. In addition to comparing ER and PR status of our patient's primary and secondary tumors, we examined an array of proteins. Pathology demonstrated positivity for established markers of breast disease, CK7 and GCDFP15, and negativity for gastrointestinal-associated proteins, CK20 and CDX-2; this constellation of results allowed for proper diagnosis as metastatic breast cancer [19, 21]. In addition to a difference in metastasis location, unique morphology of secondary tumors has been observed. ILC has been shown to metastasize diffusely, as was the case with our patient. Her course towards metastasis was likely unhindered as a result of her lack of follow through with planned treatment interventions due to a severe dependence on cocaine. Metastasis to the GI tract may manifest as intestinal wall thickening or linitis plastica if spread to the stomach [2, 6]. These lesions may be more difficult to biopsy than discrete, exophytic lesions seen in IDC [18, 20]. As such, colonoscopy may not prove diagnostic, and exploratory surgery may be required [9, 11]. Diversified approaches to diagnostic imaging including MRI may be useful in order to visualize circumferential thickening more often seen in metastatic cancer versus primary colorectal cancer [18].

Recent data show an increasing incidence of ILC, which suggests the need for increased GI surveillance among these breast cancer patients [16]. Recent studies have explored the potential efficacy of a wide range of surveillance techniques to include monitoring peripheral blood for circulating tumor cells and new ways to classify risk of future metastasis [22].

In addition to increased surveillance, early systemic treatment may also be considered in order to prevent advanced secondary disease. Some studies have recommended systemic treatment for GI metastasis, though further research is needed to properly assess a significant difference in survival outcomes [8]. Further research, both clinical and at autopsy, may further elucidate patterns in the pathogenesis of breast cancer GI metastases allowing for more effective prevention of and targeted follow-up for metastatic breast cancer.

## 4. Conclusion

Physicians and surgeons who frequently treat breast cancer, particularly with the increasing incidence of invasive lobular carcinoma must remain circumspect when evaluating a patient with known breast cancer for new gastrointestinal complaints as this may be manifestation of metastatic spread. It is prudent to consider this as the etiology for symptoms and seek to identify this with tissue biopsy and immunohistochemical staining.
